# Impact of IL-15 and latency reversing agent combinations in the reactivation and NK cell-mediated suppression of the HIV reservoir

**DOI:** 10.1038/s41598-022-23010-5

**Published:** 2022-11-03

**Authors:** Daniela Angela Covino, Maria Giovanna Desimio, Margherita Doria

**Affiliations:** Primary Immunodeficiency Research Unit, Bambino Gesù Children’s Hospital, IRCCS, 00165 Rome, Italy

**Keywords:** Immunology, Viral immune evasion

## Abstract

Inhibitors of histone deacetylases (HDACis) are major latency reversing agent (LRA) candidates in ‘shock and kill’ strategies to eradicate the HIV reservoir in infected patients. The poor achievements of initial HDACi-based trials and subsequent studies have highlighted the need for more efficient approaches such as combinatory and immunostimulating therapies. Here we studied combinations of IL-15 with pan-HDACi (Vorinostat, Romidepsin, Panobinostat) or class I selective-HDACi (Entinostat) with or without a PKC agonist (Prostratin) for their impact on in vitro reactivation and NK cell-mediated suppression of latent HIV. Results showed that pan-HDACis but not Entinostat reduced NK cell viability and function; yet, combined IL-15 reverted the negative effects of pan-HDACis except for Panobinostat. All HDACis were ineffective at reactivating HIV in a CD4^+^ T cell model of latency, with pan-HDACis suppressing spontaneous and IL-15- or Prostratin-induced HIV release, while IL-15 + Prostratin combination showed maximal activity. Moreover, Panobinostat impaired STAT5 and NF-κB activation by IL-15 and Prostratin, respectively. Finally, by using effectors (NK) and targets (latently infected CD4^+^ T cells) equally exposed to drug combinations, we found that IL-15-mediated suppression of HIV reactivation by NK cells was inhibited by Panobinostat. Our data raise concerns and encouragements for therapeutic application of IL-15/LRA combinations.

## Introduction

The introduction of an effective antiretroviral therapy (ART) has represented a major advance in the treatment of HIV infection, but a final cure is missing. A critical obstacle to overcame is persistence in ART-treated patients of latently infected cells harboring replication competent virus, which are responsible for viral rebound observed upon treatment interruption^1^. In the last decade a range of therapeutic strategies targeting the HIV reservoir has been developed. A foremost approach, called ‘shock and kill’, aims at reactivating latent HIV (shock) by using latency reversing agents (LRAs) to achieve viral reservoir elimination via immune responses and virus-mediated cytopathic effects (kill)^2^. Several classes of LRAs demonstrated HIV reactivation activity in vitro and ex vivo in CD4^+^ T cells derived from ART-treated patients, including inhibitors of histone deacetylases (HDACis), PKC agonists (PKCas) such as Bryostatin (BRY), Prostratin (PRO), and Ingenol, and P-TEFb inducers like Disulfiram and hexamethylene bisacetamine (HMBA)^3^. Three HDACis (Vorinostat/VOR, Panobinostat/PAN, Romidepsin/ROM), BRY, and Disulfiram, have been already tested in clinical trials in ART-treated individuals, but none reported strong HIV reactivation and the viral reservoir size was basically unaffected^4–7^. The failure to reduce the size of the HIV reservoir was associated not only to low virus reactivation, but also to insufficient clearance of cells that exit viral latency by the host immune responses. These earliest trials have prompted efforts to find LRAs with reduced toxicity and enhanced potency and specificity to be used in isolation or, eventually, in synergistic combinations.

Since initial attempts have focused on HDACis that act on a broad range of HDAC classes (pan-HDACis), more specific HDACis will likely enter in HIV cure strategies, with Entinostat (ENT) being a good candidate based on its selectivity for class I HDACs that promote HIV latency and large clinical application in cancer patients^8^. Moreover, new classes of drugs with both immunomodulatory and LRA properties are being investigated, which include Toll-Like Receptor agonists, immune check point inhibitors and cytokines such as IL-15^9,10^. In particular, the N-803 synthetic superagonist of IL-15 is under clinical investigation in ART-suppressed HIV^+^ patients^11^ and results of a phase 1 trial showed a modest reduction of the inducible virus in PBMCs associated with NK cell activation and expansion^12^, hence larger clinical studies are needed to firmly establish the impact of N-803 on the HIV reservoir. Notably, studies in non-human primate models demonstrated that CD8^+^ T cells suppress HIV transcription through a non-cytolytic activity that can blunt the latency reversal effect of N-803 or, as shown in vitro in human cells, other LRAs, whereas NK cells apparently lack such pro-latency activity^13–15^. This important evidence has renewed the focus on exploiting the killing capacity of NK cells to improve the efficacy of HIV cure strategies^16–18^, also galvanized by recent advances in the understanding of NK biology and development of several NK cell-based immunotherapies^19^. NK cells are important components of the innate immune system endowed with antigen-independent cytotoxicity against virus-infected cells and tumors, an activity orchestrated by the balance of opposite signals delivered by activating/inhibitory receptors upon engagement of cognate ligands on target cells; NK cells also kill targets via antibody-dependent cellular cytotoxicity and promote adaptive immunity by releasing numerous cytokines and chemokines^20^.

Since 2-drug combination strategies will likely be needed to achieve clearance of the HIV reservoir, one concern consists in the possibility that the outcome of IL-15 or other immunomodulatory drug could be hampered by the opposite effect of a second LRA used in association. Indeed, work from various laboratories including ours has shown that LRAs, mostly pan-HDACis, decrease the viability of NK and/or T cells exposed in vitro, though some results were not consistent in all studies^21–26^. Moreover, evidence has been provided that pan-HDACis and HMBA can interfere with NK cell cytotoxicity by down-regulating their activating receptors^22–29^, particularly NKG2D that is crucial for the recognition and killing of tumors and virus-infected cells^30^. Of note, the deleterious effect of ROM on NK cell cytotoxicity could be reversed through the association with PRO, a PKCa with NK-cell stimulating properties^27^. However, this was not the case for HMBA that, by reducing expression of both NKG2D and its DAP10 adaptor, impaired NK cell cytotoxicity also in the presence of PRO or IL-15^28^. One additional aspect to be considered is that many LRAs can induce expression of both latent HIV and ligands for NK cell receptors that share common regulatory mechanisms^27,31,32^, hence they have the potential to influence the type of interactions occurring between HIV^+^ T cells and NK cells.

Overall, to achieve promising curative strategies for ART-treated patients, it is critical to find LRA combinations that effectively reverse HIV latency while preserving, if not enhancing, the capacity of NK cells to clear the reactivated viral reservoir. In this study, we evaluated combinations of one HDACi, either unselective (VOR, ROM, PAN) or class I HDAC-selective (ENT), with PRO and/or IL-15 for their effects on the viability of NK and CD4^+^ T cells, on the phenotype and cytotoxic activity of NK cells, and on HIV reactivation in a CD4^+^ T cell-based experimental model of latency. At last, NK cell-mediated suppression of reactivated HIV was tested in co-cultures in which both effectors (NK) and targets (latently infected CD4^+^ T cells) have been equally exposed to IL-15/LRAs combinations.

## Results

### IL-15 and PRO can attenuate pan-HDACi toxicity on NK and CD4^+^ T cells

**Figure 1:**
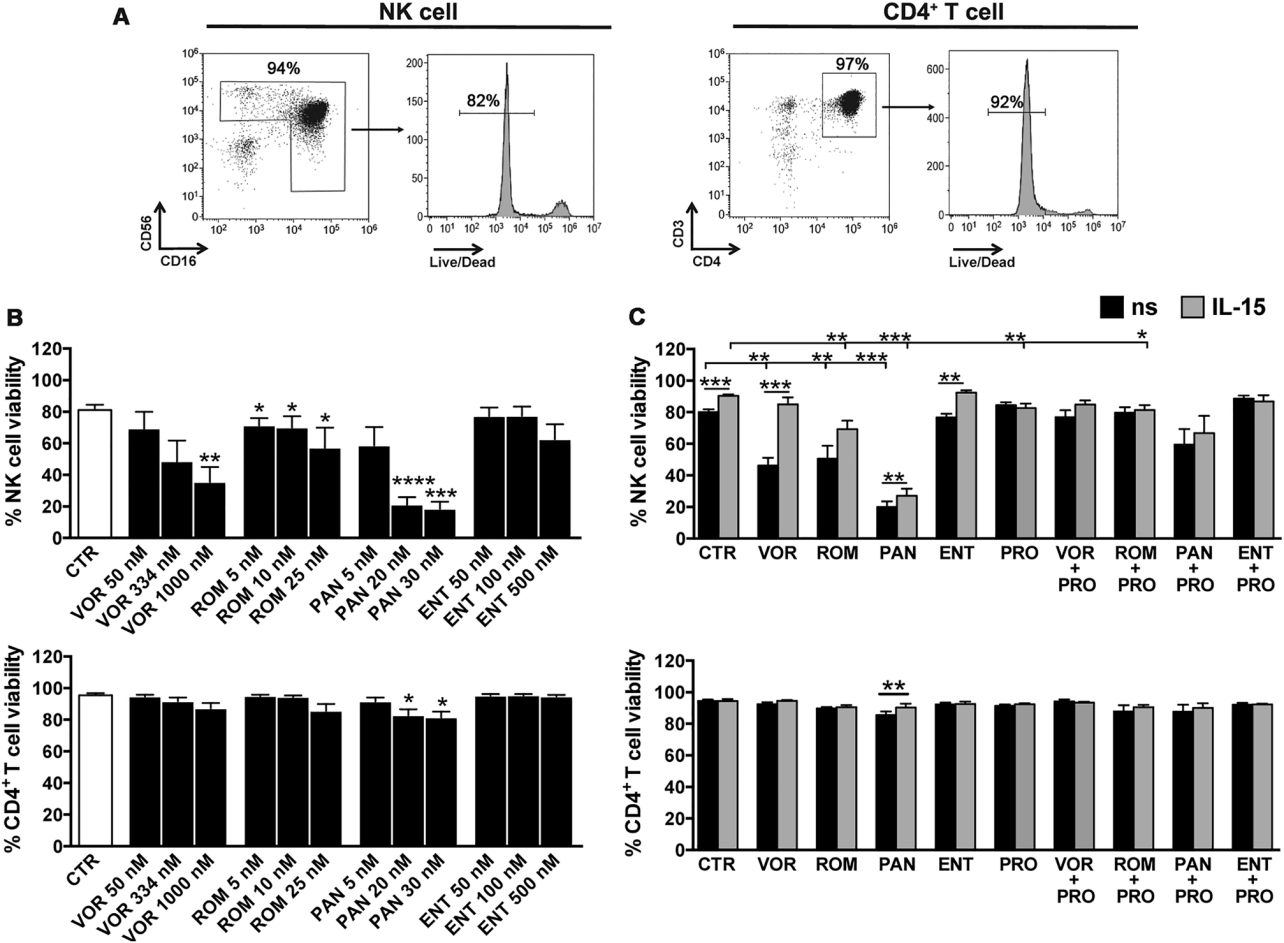
Effect of IL-15/LRA combinations on NK and CD4^+^ T cell viability*.* NK and CD4^+^ T cells purified from PBMCs of healthy donors were cultivated for 72 h without stimuli or in the presence of single or combined LRAs, then cell viability was examined by LIVE/DEAD staining. Cell viability was examined after 72 h by LIVE/DEAD staining. (**A**) Percentage of live purified NK and CD4^+^ T cells of representative unstimulated cell cultures from one donor. (**B**) NK and CD4^+^ T cells were untreated (control, CTR) or stimulated with different doses of VOR (50, 334, 1000 nM), ROM (5, 10, 25 nM), PAN (5, 20, 30 nM) or ENT (50, 100, 500 nM). Bars represent mean ± SEM obtained from 4 independent donors. Statistics was performed using paired test versus unstimulated control. (**C**) NK and CD4^+^ T cells were cultivated without stimuli (CTR), in the presence of 334 nM VOR, 20 nM PAN, 10 nM ROM, 100 nM ENT, 1 µM PRO alone or in HDACi + PRO combinations, all condition including or not 12.5 ng/ml of IL-15. Bars represent mean ± SEM obtained from at least 4 independent donors. Statistics was performed using paired Wilcoxon or *t*-test for non-parametric and parametric distributions, respectively. *p < 0.05; **p < 0.01; ***p < 0.001; ****p < 0.0001.

First, we measured by flow cytometry the viability of primary NK and CD4^+^ T cells isolated from PBMCs of healthy donors and cultivated for 72 h in the presence of HDACis at three concentrations within the range used previously for ex vivo or in vitro reactivation and observed in plasma of treated patients (Fig. 1A,B)^8,24,33–36^. We found that NK cell viability was reduced in a dose-dependent manner by VOR and ROM and, particularly, by PAN (up to 75% reduction) in line with previous work^21–23,26,28,37^, whereas ENT showed minimal toxicity at the highest concentration (500 nM). On the other hand, CD4^+^ T cell were not particularly affected by HDACis, though PAN had a slightly toxic effect at 20–30 nM concentrations. Then, we investigated whether the viability of NK and CD4^+^ T cells exposed to HDACi with and without PRO could benefit from addition of IL-15 because of its well-known pleiotropic capacity to promote cell survival^11^. In these experiments, HDACis were used at a single dose that was previously reported as clinically relevant (334 nM VOR, 10 nM ROM, 20 nM PAN, 100 nM ENT)^7,33,38,39^, while PRO and IL-15 were used at the lowest dose required for in vitro latent HIV reactivation (1 µM and 12.5 ng/ml, respectively)^40,41^. Results with NK cells showed that PRO was not toxic when used alone and had a positive effect in pan-HDACi + PRO combinations, resulting in NK cell viability similar to control cultures (Fig. 1C). The addition of IL-15, which individually enhanced the percentage of live NK cells, improved viability of HDACi-treated cells, though it was fully recovered only in VOR + IL-15 cultures, but did not further stimulate PRO and HDACi + PRO culture growth. Moreover, in CD4^+^ T cells the slightly toxic effect of PAN was abrogated by IL-15 addition.

### Impact of IL-15/LRA combinations on NK-cell phenotype and function

**Figure 2:**
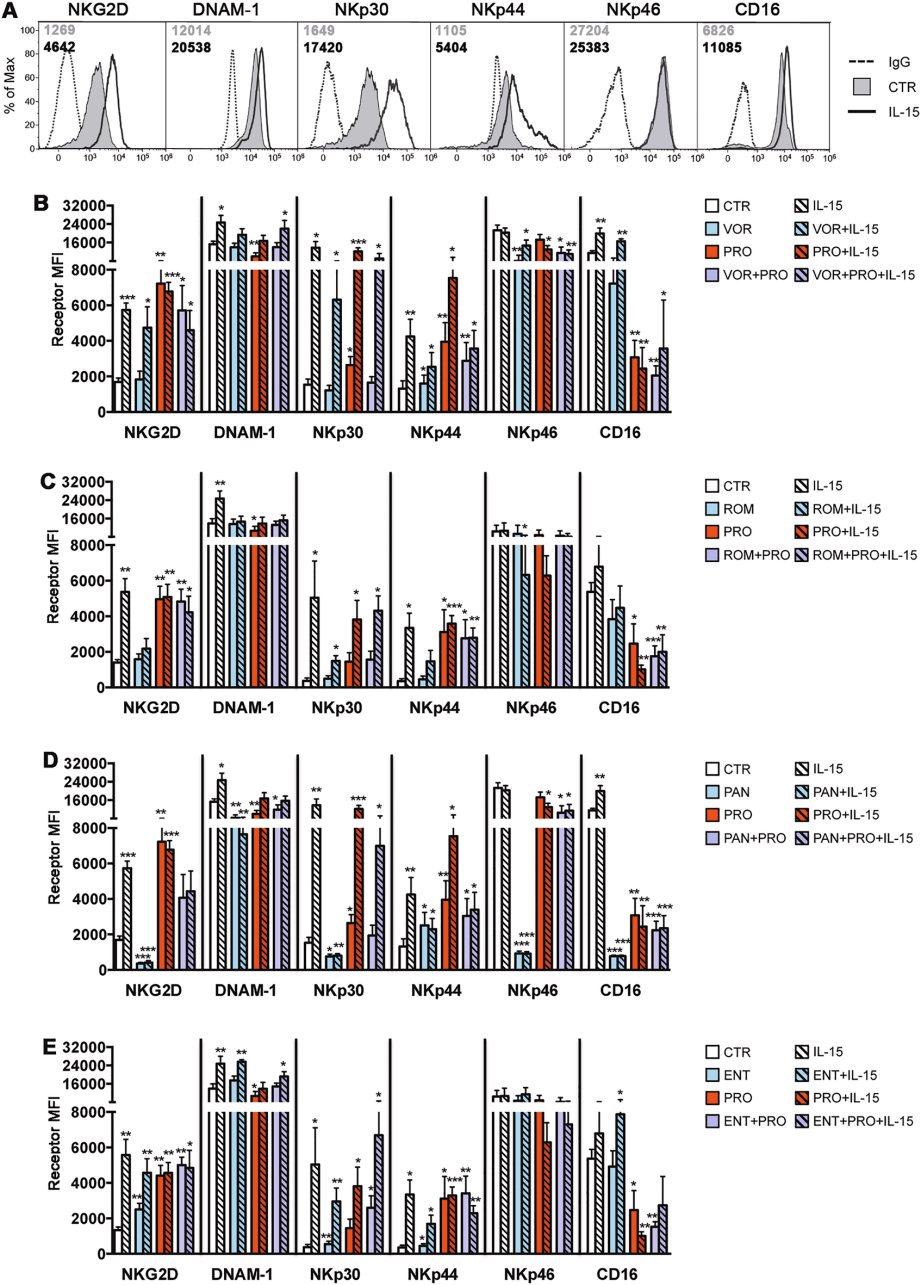
IL-15/LRA combinations variably affect NK-cell phenotype*.* Purified NK cells were cultivated for 72 h with IL-15/LRA combinations as described in Fig. 1 prior analysis of cell-surface receptors. (**A**) Histograms show representative expression of NKG2D, DNAM-1, NKp30, NKp44, NKp46, and CD16 in NK cells exposed or not to IL-15; mean fluorescence intensity (MFI) values for untreated CTR cells (filled gray) and IL-15-treated cells (solid line) together with control IgG signal (dashed line) are reported. (**B–E**) Each panel represents a set of experiments performed with at least 5 independent donors for a given HDACi used alone or in combination with IL-15 and/or PRO (individual or combined VOR, ROM, PAN, and ENT are depicted in panels (**B**), (**C**), (**D**), and (**E**), respectively). Mean ± SEM MFI is shown. Statistics was performed using paired *t*-test versus unstimulated control. *p < 0.05; **p < 0.01; ***p < 0.001.

Next, we assessed the impact of the same single and combined drug treatments of 72 h on the phenotype and function of NK cells. First, we measured the cell-surface expression of NK-cell activating receptors including NKG2D, DNAM1, NKp46, NKp44, NKp30, and CD16 (Fig. 2). As expected from previous studies^27,40^, stimulation with IL-15 resulted in up-regulation of all receptors with the exception of NKp46 (Fig. 2A), whereas activation with PRO induced up-regulation of NK2GD, NKp30, NKp44 and, simultaneously, down-regulation of CD16 via proteolytic shedding (Fig. 2B–E). While treatment with pan-HDACis either significantly reduced expression of most receptors (PAN; Fig. 2D) or had only modest effects (VOR, ROM; Fig. 2B,C), exposure to ENT instead resulted in 2-folds up-regulation of NKG2D (Fig. 2E). The addition of IL-15 to any HDACi other than PAN generally up-modulated NK cell receptors as compared to the single HDACi treatment, although the expression level was never as high as with IL-15 alone apart from DNAM1 and CD16 in the ENT + IL-15 condition. The negative effects of PAN on all receptors excluding NKp44 were not restored by IL-15 supplement but were partially attenuated by adding PRO with or without IL-15. Analogously, we observed positive effects when PRO was added to VOR, ROM or ENT, with an increase in the expression of NKG2D, NKp30 and NKp44 as compared to HDACi alone or CTR, but CD16 expression (down-modulated by PRO) was decreased. Overall, the stimulatory effects of PRO and IL-15 on NK cell activating receptors was largely maintained when used in association with any HDACi with the exception of the PAN + IL-15 combination in which the PAN-mediated downregulation of various receptors persisted. By combining IL-15 with PRO, NKp44 but not NKG2D and NKp30, was further up-modulated as compared with single treatments, whereas both NKp46 and CD16 were reduced in comparison with IL-15 or PRO alone as well as CTR. Finally, the addition of IL-15 had minor effects on receptor modulation in HDACi + PRO combinations, with the exception of NKp30 that was up-regulated to higher levels, comparable to those observed with IL-15 alone.

**Figure 3:**
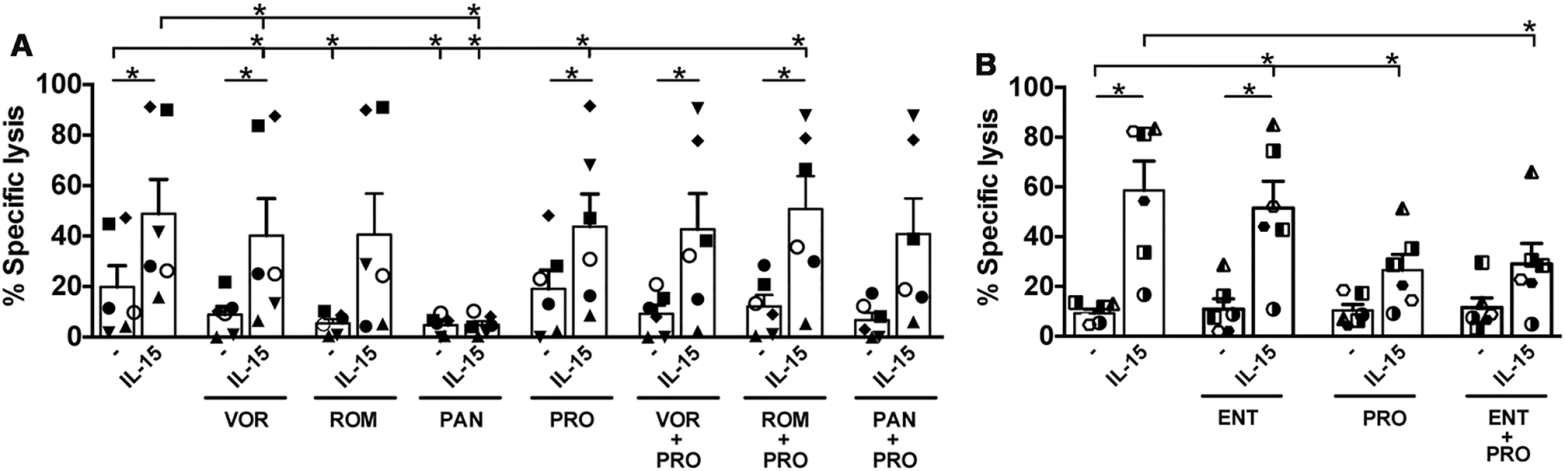
Impact of IL-15/LRA combinations on NK-cell cytoxicity. NK cells cultivated for 72 h without stimuli or with IL-15, HDACi or PRO alone or in HDACi + PRO combinations with or without addition of IL-15, were tested for cytotoxicity against K562 cell targets. The percent of specific lysis was calculated in six independent donors for pan-HDACi (**A**) as well as for ENT (**B**) combinations (each symbol represents one individual donor). The bars represent mean ± SEM. Comparisons were performed using paired Wilcoxon test versus unstimulated control, IL-15 alone, and LRA without IL-15. *p < 0.05.

To assess their cytotoxic activity after 72 h of treatment, NK cells were challenged for 4 h with K562 cells, an erythroleukemia cell line that expresses ligands for several activating receptors (NKG2D, DNAM-1, NKp30, NKp44, NKp46) and is devoid of HLA-I ligands for inhibitory receptors^42^, hence functioning as a highly susceptible target of NK cell cytotoxicity. Figure 3 shows that in most donors pan-HDACi but not ENT impaired cytotoxicity as compared with CTR. In addition, treatment with PRO had no particular effect and, when combined with any HDACi other than ENT, a trend towards inhibition was observed. As expected, exposure to IL-15 boosted cytotoxicity of NK cells in all tested donors, a positive effect that was generally maintained in IL-15 + HDACi combinations with the exception of PAN + IL-15 by which NK-cell function was strongly impaired as in the single PAN condition. Combining IL-15 with PRO had a donor-to-donor variable effect but overall did not significantly change the IL-15-mediated increase of cytotoxicity. Finally, treatment with the triple HDACi + PRO + IL-15 combinations resulted in an enhancement of NK cell cytotoxicity comparable to the PRO + IL-15 conditions without HDACi.

### Latent HIV reactivation induced by IL-15 and/or PRO is inhibited by pan-HDACis

**Figure 4:**
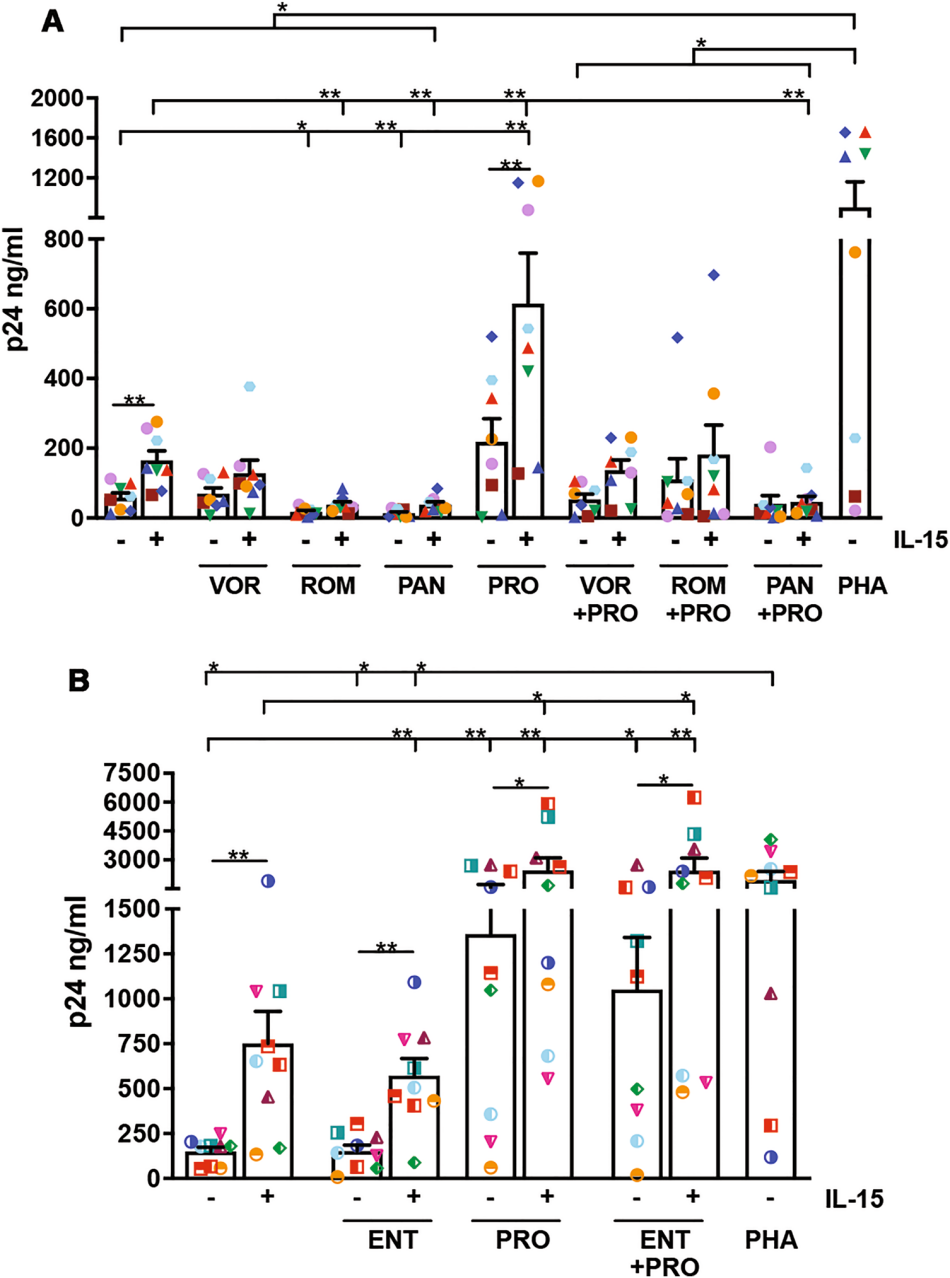
Effect of IL-15 addition to HDACi, PRO, and HDACi + PRO on reactivation of HIV in latently infected CD4^+^ T cells. Latent HIV infection was established in resting CD4^+^ T cells derived from healthy donors as described in Materials and Methods. Three days post-infection cells were collected and replaced in culture in the following conditions: medium alone or supplemented with IL-15, HDACi or PRO alone or in HDACi + PRO combinations with or without addition of IL-15, or PHA. After 48 h cells were washed and seeded at 2 × 10^6^/ml in the same initial conditions. Five days later, p24 released in the culture medium was analyzed by ELISA. Eight and nine different donors (each represented with one symbol) were tested for combinations of pan-HDACi (**A**) and ENT (**B**), respectively. Bars represent mean ± SEM. Comparisons were performed using paired Wilcoxon or *t*-test versus unstimulated control, IL-15 alone, LRA without IL-15, or PHA. *p < 0.05; **p < 0.01.

Then, IL-15/LRA combinations were tested for their capacity to revert latent HIV infection established in resting CD4^+^ T lymphocytes of healthy donors according to a standard protocol (see Materials and Methods). In this experimental system, latent provirus activation by strong stimuli, such as PRO and PHA, can be detected after 3 days by analyzing in flow cytometry the appearance of cells expressing the viral p24 Gag capsid antigen (Supplementary Fig. S1); in order to quantitatively measure HIV reactivation also in suboptimal stimulatory conditions, latently infected T cells cultivated for 48 h in medium alone or supplemented with single and combined IL-15/LRAs or PHA (maximal stimulation) were collected, resuspended at 2 × 10^6^/ml in fresh medium with the same initial supplementation, and further cultivated for 5 days till when the amounts of newly produced HIV particles were measured in the culture supernatant by p24 ELISA. A total of 8 and 9 independent donors were tested in experiments performed with pan-HDACis (Fig. 4A) and ENT (Fig. 4B), respectively. Results showed that not only HDACis used individually were unable to induce latent HIV reactivation but also that ROM and PAN significantly reduced spontaneous viral reactivation measured in unstimulated cultures (17.54 ± 4 and 13.4 ± 4, respectively, vs 58 ± 13 p24 ng/ml, mean ± SEM). These data were confirmed over the range of HDACi concentrations used in previous in vitro studies and shown in Fig. 1B (Supplementary Fig. S2). On the other hand, IL-15 enhanced HIV reactivation over the basal spontaneous levels in all but one tested donor (from 1.25 to 13.6-fold increase). The response to PRO stimulation was highly donor-dependent since we found no effect on HIV reactivation in 4 out of 17 donors as well as an increase in HIV release ranging from 1.4 to 35.8 folds in those individuals who responded. When IL-15 and PRO were administered together, HIV release was always above the level found in untreated cultures, reaching very high levels in 9 donors, similar or higher if compared to those achieved with PHA stimulation. In HDACi + IL-15 combinations, the stimulatory effect of IL-15 was abrogated when used in association with any pan-HDACi but only slightly reduced when combined with ENT, with HIV release being significantly higher in ENT + IL-15-treated as compared with unstimulated cultures (572.7 ± 95.5 vs 150 ± 23.8 p24 ng/ml). Additionally, HIV reactivation by PRO and PRO + IL-15 was repeatedly inhibited when a pan-HDACi was also included, whereas it was maintained at similarly high levels if ENT was added.

### PAN interferes with IL-15 and PRO stimulation

**Figure 5:**
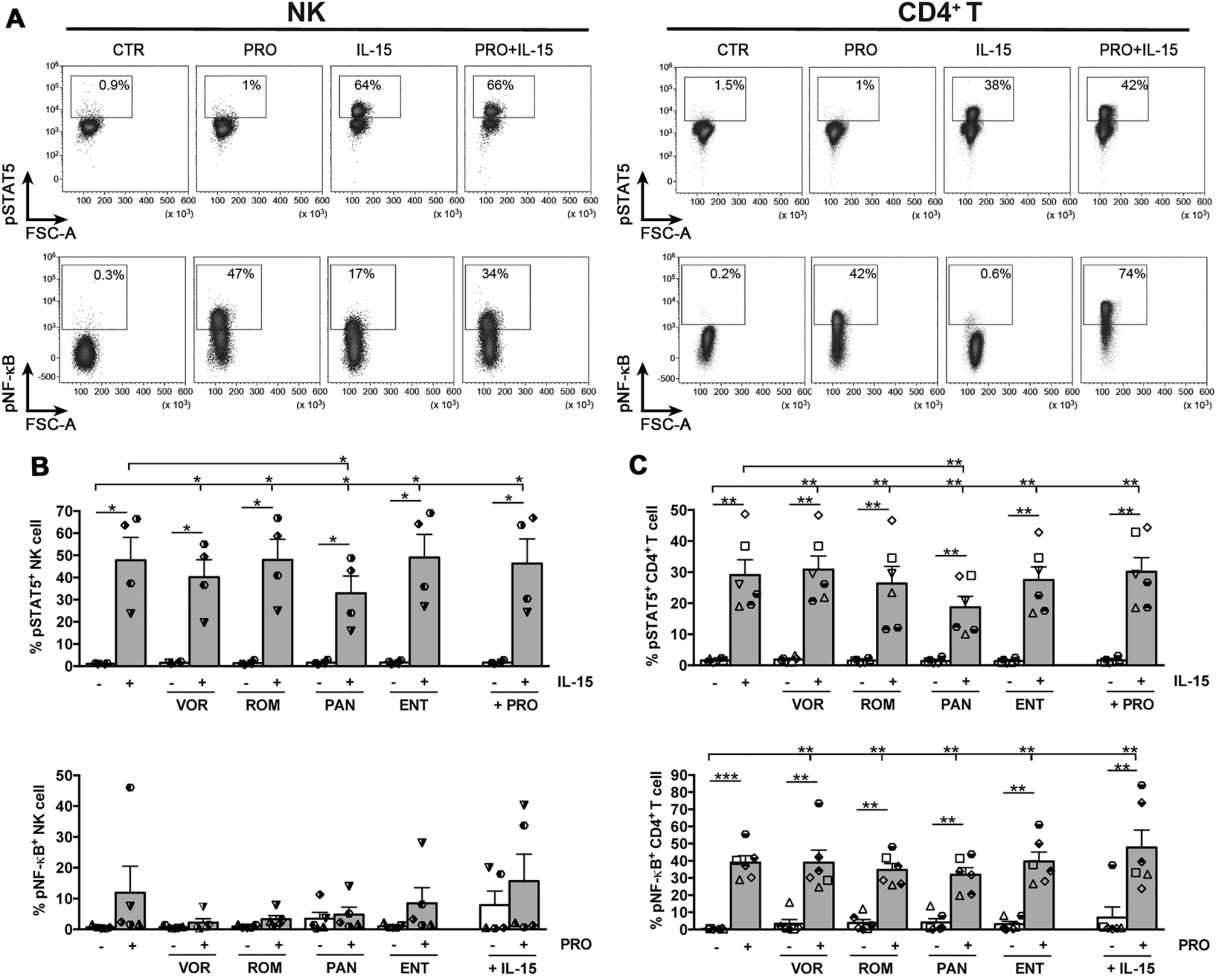
Induction of pSTAT5 and pNF-κB^+^ in IL-15/LRA treated NK and CD4^+^ T cells. Purified NK or CD4^+^ T cells were cultivated overnight in serum-free medium without stimuli (CTR) or in the presence of VOR, PAN, ROM, or ENT. Then, cells were incubated at 37 °C for 15 min in fresh medium alone or containing 1 µM PRO and/or 12.5 ng/ml IL-15 and immediately fixed/permeabilized and analyzed for pSTAT5 and pNF-κB expression by FACS. (**A**) Dot plots show percentage of pSTAT5^+^ and pNF-κB^+^ cells among NK and CD4^+^ T cells in CTR, PRO, IL-15 or PRO + IL-15 conditions for a representative experiment. (**B, C**) Bars depict mean ± SEM percentages of pSTAT5^+^ and NF-κB^+^ NK cells (**B**, n = 4–5) and T cells (**C**, n = 6). Each symbol represents one donor. Statistics was performed using paired *t*-test versus unstimulated control, IL-15 alone, and LRA without IL-15. *p < 0.05; **p < 0.01.

While carrying out this study we found that pan-HDACis, particularly PAN, can interfere with IL-15 capacity to stimulate cytotoxicity of NK cells and reactivation of latent HIV in CD4^+^ T cells; in addition, pan-HDACis can inhibit the induction of HIV reactivation by PRO. To understand the molecular basis of these detrimental drug interactions, we cultivated purified NK and T cells for 18 h in serum-free medium with or without HDACi, then cells were stimulated or not for 15 min with IL-15 and/or PRO and analyzed by flow cytometry to measure nuclear accumulation of a major downstream effector molecule, namely phosphorylated STAT5 (pSTAT5) and NF-κB (pNF-κB) for IL-15 and PRO stimulation, respectively (Fig. 5A). IL-15 potently induced pSTAT5 and this effect was slightly reduced by VOR and ROM in NK and CD4^+^ T cells, respectively, and significantly inhibited by PAN in both cell types, whereas addition of ENT or PRO had no effect in NK or CD4^+^ T cells (Fig. 5B,C). Analogously, in CD4^+^ T cells PRO efficiently stimulated pNF-κB and the presence of PAN decreased this effect in 4 out of 6 donors analyzed, whereas the addition of other HDACi or IL-15 had no significant effect (Fig. 5C). As shown previously^40^, the response of NK cells to PRO stimulation was highly donor-dependent, being detected only in 2 out of 5 donors, and inhibition by any HDACi was evident only in the donor with a high percentage (48%) of PRO-induced pNF-κB^+^ cells (Fig. 5B). The same NK-cell response was observed also following PMA + Ionomycin stimulation (data not shown), hence indicating an as yet inexplicable inter-individual variability in the pNF-κB activation pathway of NK cells.

In summary, PAN decreased IL-15-mediated STAT5 activation in both NK and CD4^+^ T cells and, in most donors, also reduced NF-κB activation by PRO in CD4^+^ T cells.

### IL-15-mediated suppression of HIV reactivation by NK cells can be impaired by PAN

**Figure 6:**
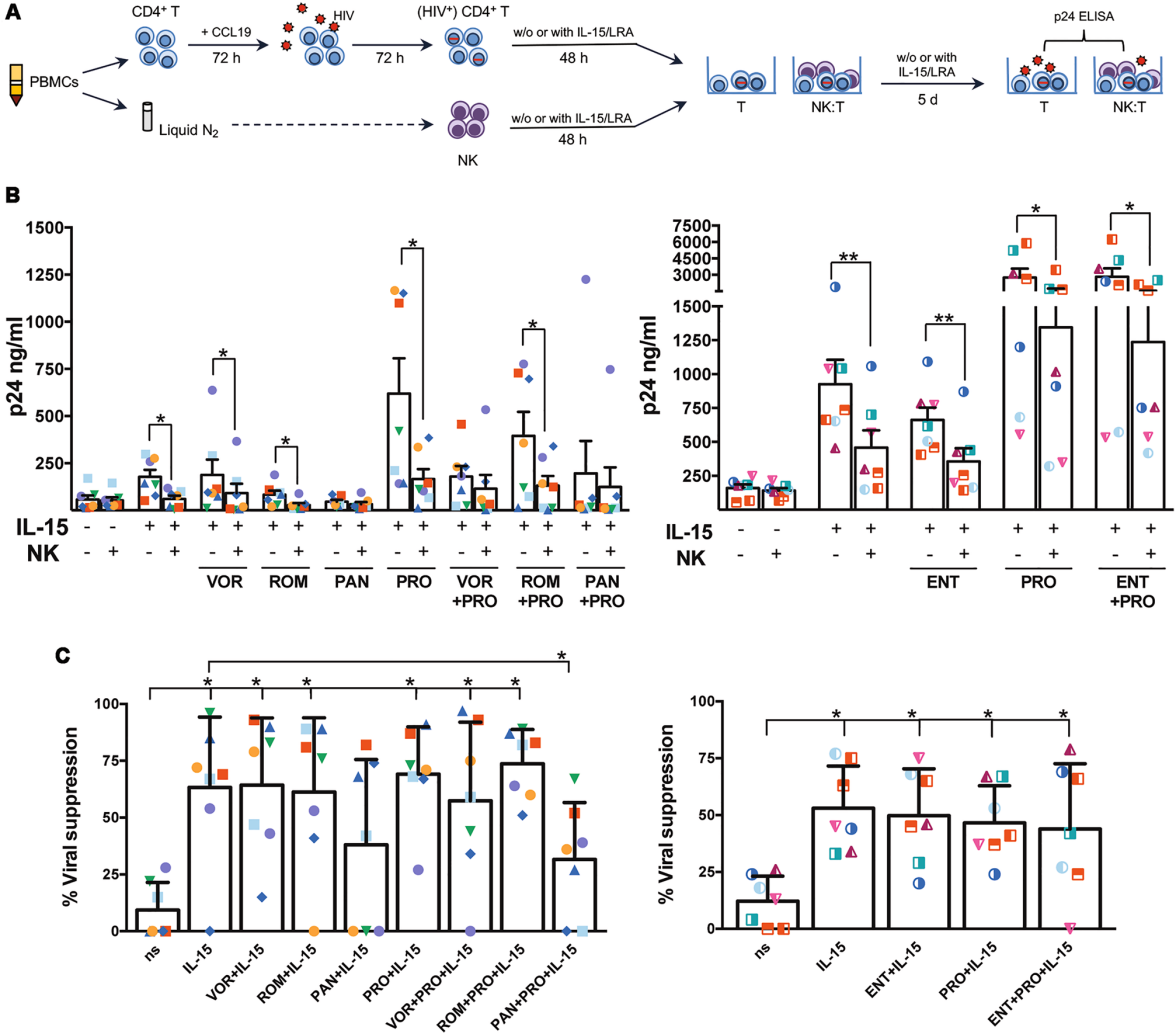
NK-cell mediated suppression of HIV reactivation in IL-15/LRA-exposed NK/T co-cultures. (**A**) Schematic experimental model. PBMCs were isolated from peripheral blood of healthy donors and used to purify CD4^+^ T cells then cultivated with 29 nM CCL19, whereas a part of cells was cryopreserved. After 72 h, CD4^+^ T cells were infected with HIV and further cultivated for 72 h in the absence of stimuli. Then, latently infected CD4^+^ T as well as NK cells purified from an aliquot of cryopreserved PBMCs of the same donors were cultivated separately in the absence of stimuli, with IL-15 (12.5 ng/ml) alone or with HDACi, PRO, or HDACi + PRO combinations. After 48 h cells were collected, washed, and CD4^+^ T cells were placed back in culture either alone or together with autologous NK cells cultivated in the same conditions at 1:1 ratio, and initial stimuli were added again. After 5 days, p24 released in the culture medium was analyzed by ELISA. (**B**) Each symbol represents one donor in the presence or absence of NK cells in the indicated conditions (untreated, IL-15 alone or with HDACi, PRO or HDACi + PRO). (**C**) The NK-cell mediated killing was expressed as percentage of viral (p24/ml) suppression. Bars represent mean ± SEM values obtained in experiments with 14 different donors (7 for pan-HDACi and for ENT combinations in right and left panels, respectively). *p < 0.05; **p < 0.01 by Wilcoxon test.

Our results indicated that IL-15 on one hand boosts NK cell cytotoxicity and, on the other hand, strongly induces HIV latency reversal in T cells especially when used in association with PRO, and that HDACi addition either have no effect (ENT) or inhibited (pan-HDACi) such IL-15 properties. Therefore, we tested whether these drug interactions may ultimately influence the capacity of NK cells to suppress the reactivation of latent HIV when both effectors and targets are simultaneously exposed IL-15/LRA combinations. To this end, after having established latent HIV infection in primary CD4^+^ T cells, we purified NK cells from aliquots of cryopreserved PBMCs of the same donors and separately cultured for 48 h both T and NK cells without stimuli, with IL-15 alone or combined with PRO, HDACi or PRO + HDACi; then, we collected all cells, plated latently infected T cells either with or without autologous NK cells pre-exposed to the same treatments at a 1:1 ratio, further stimulated the cultures with the same initial conditions and finally measured by p24 ELISA the amounts of HIV released in the medium after 5 days (the experimental procedure is depicted in Fig. 6A). Figure 6B shows raw data obtained in two sets of experiments including either pan-HDACi or ENT combinations, each one performed with 7 independent donors; the HIV-suppressive activity of NK cells was calculated as the percentage of reduced viral amount in cultures of T cells alone as compared to co-cultures with NK cells exposed to the same treatments (Fig. 6C). In all tested donors, the capacity of NK to suppress HIV spontaneously released in unstimulated cultures was modest (10 ± 3%) but strongly increased in IL-15-stimulated cultures (58 ± 7%). A similarly high viral suppression by NK cells was found in cultures exposed to IL-15 in association with PRO, HDACi or PRO + HDACi, with the sole exception of PAN that significantly inhibited the anti-viral activity of NK cells when added to IL-15 or IL-15 + PRO (Fig. 6C).

## Discussion

An ideal combination therapy to eradicate the HIV reservoir should be not only devoid of any toxic effects and efficacious at reversing viral latency, but also capable of promoting antiviral immune responses including those of NK cells. Towards this goal, here we investigated combinations of members of two major classes of LRA candidates, HDACi and PKCa, with IL-15. Actually, IL-15 and its derivatives have very promising application because of their latency-reversing potential in vitro^43^ and, at least in the absence of CD8^+^ T cells, in vivo^14^, coupled with the major biological role of IL-15 in the proliferation and survival of different lymphocytic lineages and, especially, its nonredundant role in NK-cell development, maintenance, and function^11^.

First, we showed that pan-HDACis but not ENT affected NK cell viability, particularly PAN that also down-regulated most NK cell activating receptors and reduced the cytotoxic activity of unstimulated NK cells towards tumor targets (K562), which is generally in agreement with previous work^22,24,27,37^. The addition of IL-15, a physiologic activator of NK cells, largely reverted the negative effects of VOR and ROM but not those of PAN that actually abrogated the NK cell response to IL-15 stimulation. The addition of PRO, which activates NK cells in a non-specific manner without increasing significantly K562 killing^22,40^, could contrast the negative effect on NK cell viability of any pan-HDACi as well as receptor down-regulation by PAN. Furthermore, by adding simultaneously IL-15 and PRO we observed that IL-15-mediated stimulation of cytotoxicity was maintained in the presence of all HDACis including PAN. Therefore, PKC activation in NK cells may prevail over apoptotic pathways induced by pan-HDACis, as suggested earlier^27,44^, and also allows NK cells to respond to IL-15 stimulation even in the presence of these drugs. Also, our results confirmed that exposing NK cells to ENT preserved cell viability and increased NKG2D levels, although we did not observe any increment in cytotoxicity as reported in previous studies using the same drug concentration but tumor targets other than K562 cells^29,45^; we speculate that the stimulating effect of ENT on NK cell cytotoxicity might depend on the nature of cell targets, eventually unveiled against cancer cells expressing very high levels of NKG2D ligands^29,45^.

When primary CD4^+^ T cells were exposed to HDACis, either alone or in 2/3-drug combinations with PRO and IL-15, their viability did not significantly change compared to untreated controls. By using a primary CD4^+^ T cell-based model of latency, we found that any tested HDACi was devoid of latency reversal activity whereas, as expected, IL-15 and PRO induced HIV reactivation in most donors. Moreover, our results showed that pan-HDACis but not ENT hindered spontaneous viral reactivation as measured in non-stimulated T cell cultures (ROM and PAN, particularly) and inhibited HIV reactivation by IL-15 and PRO when these latter were also present either individually or together, with PAN showing the strongest antagonisms. Remarkably, we also found that the combination of IL-15 + PRO displayed an outstanding latency reversal activity, in most donors outperforming the effect of PHA that, so far, has been considered one of the maximal HIV inducers. The here observed inefficacy of HDACis at reactivating HIV is in agreement with various studies^46–49^ but also in apparent contrast with other reports showing HDACi-induced latency reversal^8,35,50–52^, though different results can be explained by differing study designs. Herein, we did not use T cell expanding, differentiating, or pro-survival procedures that might have contrasted pro-apoptotic effects of HDACis in previous studies. In addition, we evaluated the production of p24 HIV protein rather than accumulation of HIV transcripts because these latter may not be fully processed and competent for translation of viral proteins, as recently shown for both pan- and class I selective-HDACis^43,46^, hence may not measure complete latency reversal. While HDACi + IL-15 combinations have not been previously examined, studies in which HDACi were combined with PKCas to reactivate latent HIV have produced conflicting results showing either co-operation/synergism^47,53–55^ or antagonism^8,49,51^, which again might be related to differences in the experimental procedures.

Moreover, we provided evidence for the inhibitory potential of PAN by showing that this drug significantly reduced STAT5 activation by IL-15 in NK and CD4^+^ T cells as well as PRO-induced activation of NF-κB in CD4^+^ T cells. Both pSTAT5 and pNF-κB are key transactivators of genes that promote survival and activation of NK and T cells and also induce HIV transcription^41,56,57^. The unique capacity of PAN among HDACis to interfere with signaling cascades triggered by IL-15 or PRO could be explained by its strong activity towards class II HDAC6 that, conversely, is poorly affected by VOR or ROM^35^ and spared by ENT^8^. Indeed, STAT5 activation occurs via deacetylation of its HMGN2 co-factor by HDAC6^58^; besides, to activate NF-κB, PKCas targets the HSP90/IKK complex that is maintained through HDA6-mediated deacetylation of HSP90^59,60^.

Finally, we demonstrated that the capacity of NK cells to suppress the release of HIV by autologous latently infected CD4^+^ T cells was strongly boosted by IL-15 and that this stimulation was maintained if IL-15 was used in association with PRO, HDACi or PRO + HDACi, with the exclusion of PAN that significantly inhibited the anti-viral activity of IL-15-stimulated NK cells when added alone or together with PRO. These results confirmed the impact of IL-15/LRA combinations in the viability and function of NK cells with one exception: as opposed to the K562 killing assay, PAN inhibited IL-15-mediated suppression of reactivated HIV also in the presence of PRO, suggesting that the PKCa could not fully rescued some NK cell features impaired by PAN that are important for killing HIV^+^ T cells. Interestingly, in NK cells treated with IL-15 + PAN + PRO, NKG2D and DNAM-1 were not up-modulated as with IL-15 alone and, in addition, expression of NKp46 was significantly reduced as compared to IL-15-stimulated or untreated cells (Fig. 2D). These results indicate that PAN interferes with the expression and function of receptors that are key for the capacity of activated NK cells to recognize and kill HIV^+^ T cell targets, hence confirming the important role of NKG2D, DNAM-1, and NKp46 in NK-cell responses against HIV^61–65^.

Triple combinations (HDACi + PKCa + IL-15) have been instrumental here to study drug interactions despite their clinical application is not under consideration. On the other hand, new trials combining HDACi with a distinct therapeutic product have been approved (VOR with Tamoxifen, HIV-specific T cells, or HIV vaccine and PAN with pegylated IFNα; NCT03382834, NCT03212989, NCT02336074, NCT02471430, respectively), and will likely continue to be developed. In this regard, our results raise concerns for the application of PAN in HIV cure strategies, even if combined with IL-15, because of its antagonistic effects in HIV latency reversal as well as in anti-viral NK cell function. Besides, combining IL-15 with VOR, ROM or ENT did not seem to confer any advantage in terms of HIV reactivation or NK cell-mediated suppression as compared with the sole IL-15 treatment. Conversely, IL-15 + PRO represents a very promising combination therapy considering that the efficiency of HIV reactivation by IL-15 was potently boosted by PRO, likely trough the synergism of pSTAT5 and pNF-κB and, possibly, other simultaneously activated inducers of viral transcription.

Using different experimental design, some previous works showed that NK cell-mediated killing of p24^+^ T cells that exit from latently was not affected by pan-HDACis but stimulated by PRO^40^. Additionally, Garrido et al*.* showed that prior stimulation with IL-15 enabled NK cells to effectively suppress HIV outgrowth when joined to latently infected T cell cultures pre-exposed to VOR^66^. Here we added novel information by setting up a viral suppression assay in which effectors and target cells have been exposed to the same drugs for an equal period of time, thus reflecting the conditions that may occur in a clinical setting.

We realize that a limitation of this study consists in the absence of ex vivo tests, which was due to insufficient availability of cells from ART-treated patients. It is worth mentioning, though, that there is so far a broad correspondence between in vitro and ex vivo latency studies for what regards responses to LRAs or IL-15.

Overall, the present study provides evidence that administration of PAN, even if combined with IL-15, is unlikely to be effective in both the ‘shock’ and ‘kill’ phases of an HIV eradication strategy. Moreover, we identified IL-15 + PRO as a novel successful combination therapy in terms of latent HIV reactivation and clearance by NK cells. Notably, novel PKCas with more tolerable levels of toxicities and improved LRAs capacity compared with PRO are continuously being developed^67,68^ and offer great potential for optimizing IL-15-encompassing strategies towards the elimination of the viral reservoir in people infected with HIV.

## Materials and methods

### Cells, antibodies, and reagents

PBMCs were obtained by Ficoll separation of buffy coats from the donor bank of the Bambino Gesù Children’s Hospital. Use of buffy coat was approved by the Ethical committee of the Bambino Gesù Children’s Hospital and written informed consent from all participants was obtained, in accordance with the Declaration of Helsinki. Primary NK and CD4^+^ T cells were purified from PBMCs with 90–98% purity by negative selection with cell-type specific EasySepEnrichment Kit (Stem Cell Technologies) according to manufacturer’s protocol. Primary cells were maintained in complete RPMI 1640 medium supplemented with 10% fetal bovine serum, 0.2 mM L-glutamine (all from Gibco/Thermo Fisher Scientific, MA, USA) and 100 units/ml penicillin–streptomycin (Euroclone, Italy). The purity (~ 95%) of isolated NK (CD3^−^CD56^+^CD16^−/+^) and CD4^+^ T cells (CD3^+^CD4^+^) was assessed by immunolabeling and FACS analysis.

For flow cytometry, isotype control IgG, (BD Pharmingen, CA, USA) and the following mouse monoclonal antibodies (mAbs) were used: CD3/AlexaFluor700 (UCHT1), CD56/PerCpCy5.5 (B159), CD16/BV510 (3G8), pNF-κB (S529)/Alexa Fluor 647 and pSTAT5 (Y694)/Alexa Fluor 488 from BD Pharmingen; NKG2D(CD314)/BV785 (1D11), DNAM-1(CD226)/FITC (11A8), NKp30/APC , NKp44/PE (P44-8), NKp46/PE-Cy7 (9E2), from BioLegend (CA, USA); p24/FITC (KC57) from Beckman Coulter (CA, USA). Where indicated, cells were treated with 335 nM suberoylanilide hydroxamic acid (Vorinostat, VOR), 10 nM Romidepsin (ROM), 20 nM Panobinostat (PAN), 100 nM Entinostat (MS-275, ENT), 1 µM Prostratin (PRO), 10 µg/ml phytohemagglutinin (PHA) or with equivalent amounts of dimethyl sulfoxide (DMSO) when used as a solvent (all from Sigma-Aldrich, MO, USA with the exception of ENT from Selleckchem, TX, USA). Lower and higher doses of HDACis were used in some experiments to test dose-dependent relationships. Other reagents used were: CCL19 (R&D Systems), IL-15 (Peproteck, UK), 5,6-carboxyfluorescein diacetate succinimidyl ester (CFSE) and 7-aminoactinomy-cin D (7-AAD), both from Sigma-Aldrich.

### Flow cytometry

To assess viability, cells were stained with LIVE/DEAD fixable NEAR-IR dead cell stain kit according to manufacturer’s protocol (Invitrogen, Thermo Fisher Scientific). To label NK cell-surface receptor, cells were incubated for 20 min at 4 °C with specific mAbs. For detection of intracellular p24, latently HIV-infected CD4^+^ T cells were fixed with 1% paraformaldehyde (PFA) for 10 min, permeabilized with Permeabilizing Solution 2 of BD Biosciences reagents for 10 min, then incubated for 30 min with p24/FITC mAb; all steps were performed at room temperature.

For pNF-κB and pSTAT5 analysis, purified NK and CD4^+^ T cells were resuspended in serum-free medium supplemented or not with HDACi (334 nM VOR, 10 nM ROM, 20 nM PAN or 100 nM ENT), plated in quadruplicates in 96-well plate and cultivated at 37 °C overnight. Then, for each quadruplicate, medium alone or containing IL-15, PRO, or IL-15 + PRO, was added to each of the four replicates to reach final concentration of 12.5 ng/ml IL-15 and 1 µM PRO and cells incubated at 37 °C for 15 min. Cells were then fixed (10 min, 37 °C) with the Cytofix Fixation Buffer (BD Biosciences, 554655), permeabilized (10 min, on ice) with the ice-cold Permeabilization Buffer III (BD Biosciences, 558050). Finally, cells were stained (30 min, RT) with anti-pNF-κB (S529) and anti-pSTAT5 (Y694) antibodies.

All immunolabeled cells resuspended in 1% paraformaldehyde (PFA) were acquired on Cytoflex (Beckman Coulter) or on a FACSCanto II (BD Biosciences). Positive cell gating was set using fluorescence minus one control (FMO). Mean fluorescence intensity (MFI) was subtracted of the value obtained with isotype control antibody. Data analyses were performed using Kaluza (Beckman Coulter) or FlowJo v10 (BD Biosciences).

### NK-cell cytotoxicity assays

Flow cytometry-based cytotoxicity assays were performed using K562 cells as targets (T) and primary NK cells as effectors (E) previously cultivated for 72 h in medium alone or supplemented IL-15 (12.5 ng/ml) and containing or not HDACi (334 nM VOR, 10 nM ROM, 20 nM PAN, 100 nM ENT), PRO (1 µM), or HDACi + PRO. K562 cells were labelled with 2.5 μM CFSE for 7 min at 37 °C, washed twice, then 2.5 × 10^5^ cells were seeded with NK cells at E:T ratio of 1:1 in a 96-well plate for 4 h at 37 °C. Then, cells were labelled with 5 μg/ml 7-AAD for 20 min at 4 °C, washed, and fixed with 1% PFA and analyzed by FACS. The percentage of specific lysis of target cells (gated as CFSE^+^) was calculated as follows: 100 × (% 7-AAD^+^ target cells in sample − basal % 7-AAD^+^ target cells)/(100 − basal % 7-AAD^+^ target cells).

### Establishment and reactivation of latently infected CD4^+^ T cells

Resting CD4^+^ T cells cultures latently infected with HIV-1 were established and then reactivated as previously described with some modifications^40^. Briefly, purified CD4^+^ T cells cultivated with 29 nM CCL19 for 1–3 days were infected by spinoculation with 300 ng p24/10^6^ cells of NL4-3 HIV-1 (NIH AIDS Reagent Program) pseudotyped with vesicular stomatitis virus glycoprotein (VSV-G) at 1200 g for 2 h, washed, and placed back in culture in complete medium alone at 5 × 10^6^/ml cell concentration. At day 3 post-infection, latently infected CD4^+^ T cells were collected and seeded at 3 × 10^6^/ml in complete medium alone or containing an HDACi (335 nM VOR, 10 nM ROM, 20 nM PAN or 100 nM ENT), 1 µM PRO, or HDACi + PRO combinations and supplemented or not with 12.5 ng/ml IL-15; also, a culture with 10 ug/ml PHA was set for maximal HIV reactivation. After 48 h of culture, cells were washed and reseeded at 2 × 10^6^/ml in the same initial conditions; finally, the intracellular p24 accumulation in T cells was analyzed by FACS and concentration of p24 in the culture medium was measured by ELISA (INNOTEST HIV Antigen mAb; FUJIREBIO, Japan) according to manufacturer’s protocol, 18 h and 5 days after the second stimulation, respectively.

### NK cell-mediated suppression of reactivated HIV

Three days after latent HIV infection of CD4^+^ T cells (T), NK cells were purified from an aliquot of cryopreserved PBMCs of the same donor (E) and both cell populations were separately cultivated in the same conditions (unstimulated, stimulated with IL-15, PHA, HDACi and PRO either alone or combined and with or without IL-15). Fourty eight hours later, 0.15–2 × 10^5^ T cells were transferred in new wells either alone and together with equally stimulated autologous E cells at an E:T ratio of 1:1 (final T cell concentration was set at 2 × 10^6^/ml in all conditions) and restimulated. After 5 days, cell culture supernatant was harvested and analyzed by p24 ELISA to measure HIV concentration. The percent of NK cell-mediated viral suppression was calculated with the following formula: 100 × (p24^+^ ng/ml cells in targets − p24^+^ ng/ml cells in targets with effectors)/(p24^+^ ng/ml cells in targets).

### Statistical analysis

All experiments have been performed independently at least three times. GraphPad Prism v6.0 software (San Diego, CA, USA) was used to perform all statistical analyses. A value of *p* < 0.05 was considered statistically significant.

## Supplementary Information

Supplementary Figure S1.

Supplementary Figure S2.

## Data Availability

The raw data generated during the present study will be made available by the corresponding authors on reasonable request.

## References

[1] Deeks SG (2021). Research priorities for an HIV cure: International AIDS Society Global Scientific Strategy 2021. Nat. Med..

[2] Kim Y, Anderson JL, Lewin SR (2018). Getting the "Kill" into "Shock and Kill": Strategies to eliminate latent HIV. Cell. Host Microbe.

[3] Rodari A, Darcis G, Van Lint CM (2021). The current status of latency reversing agents for HIV-1 remission. Annu. Rev. Virol..

[4] Archin NM (2012). Administration of vorinostat disrupts HIV-1 latency in patients on antiretroviral therapy. Nature.

[5] Elliott JH (2015). Short-term administration of disulfiram for reversal of latent HIV infection: a phase 2 dose-escalation study. Lancet HIV.

[6] Rasmussen TA (2014). Panobinostat, a histone deacetylase inhibitor, for latent-virus reactivation in HIV-infected patients on suppressive antiretroviral therapy: A phase 1/2, single group, clinical trial. Lancet HIV.

[7] Søgaard OS (2015). The depsipeptide romidepsin reverses HIV-1 latency in vivo. PLoS Pathog..

[8] Zaikos, T. D., Painter, M. M., Sebastian Kettinger, N. T., Terry, V. H. & Collins, K. L. Class 1-selective histone deacetylase (HDAC) inhibitors enhance HIV latency reversal while preserving the activity of HDAC isoforms necessary for maximal HIV gene expression. *J. Virol.* **92**, e02110-17. 10.1128/JVI.02110-17 (2018). 10.1128/JVI.02110-17 PMC5827401 29298886

[9] Kula-Pacurar A, Rodari A, Darcis G, Van Lint C (2021). Shocking HIV-1 with immunomodulatory latency reversing agents. Semin. Immunol..

[10] Singh, V., Dashti, A., Mavigner, M. & Chahroudi, A. Latency reversal 2.0: Giving the immune system a seat at the table. *Curr. HIV/AIDS Rep.* **18**, 117–127 (2021). 10.1007/s11904-020-00540-z PMC7985101 33433817

[11] Harwood O, O'Connor S (2021). Therapeutic potential of IL-15 and N-803 in HIV/SIV infection. Viruses.

[12] Miller JS (2022). Safety and virologic impact of the IL-15 superagonist N-803 in people living with HIV: A phase 1 trial. Nat. Med..

[13] Cartwright EK (2016). CD8(+) lymphocytes are required for maintaining viral suppression in SIV-infected macaques treated with short-term antiretroviral therapy. Immunity.

[14] McBrien JB (2020). Robust and persistent reactivation of SIV and HIV by N-803 and depletion of CD8(+) cells. Nature.

[15] McBrien JB (2020). Combination of CD8β depletion and Interleukin-15 superagonist N-803 induces virus reactivation in simian-human immunodeficiency virus-infected, long-term ART-treated rhesus macaques. J. Virol..

[16] Alrubayyi A, Ogbe A, Moreno Cubero E, Peppa D (2020). Harnessing natural killer cell innate and adaptive traits in HIV infection. Front. Cell. Infect. Microbiol..

[17] Sun Y, Zhou J, Jiang Y (2022). Negative regulation and protective function of natural killer cells in HIV infection: Two sides of a coin. Front. Immunol..

[18] Board NL, Moskovljevic M, Wu F, Siliciano RF, Siliciano JD (2021). Engaging innate immunity in HIV-1 cure strategies. Nat. Rev. Immunol..

[19] Wolf NK, Kissiov DU, Raulet DH (2022). Roles of natural killer cells in immunity to cancer, and applications to immunotherapy. Nat. Rev. Immunol..

[20] Campbell KS, Hasegawa J (2013). Natural killer cell biology: An update and future directions. J. Allergy Clin. Immunol..

[21] Clutton G (2016). The differential short- and long-term effects of HIV-1 latency-reversing agents on T cell function. Sci. Rep..

[22] Garrido C (2016). HIV latency-reversing agents have diverse effects on natural killer cell function. Front. Immunol..

[23] Jones RB (2014). Histone deacetylase inhibitors impair the elimination of HIV-infected cells by cytotoxic T-lymphocytes. PLoS Pathog..

[24] Pace M (2016). Histone deacetylase inhibitors enhance CD4 T cell susceptibility to NK cell killing but reduce NK cell function. PLoS Pathog..

[25] Rossi LE (2012). Histone deacetylase inhibitors impair NK cell viability and effector functions through inhibition of activation and receptor expression. J. Leukoc. Biol..

[26] Walker-Sperling VE, Pohlmeyer CW, Tarwater PM, Blankson JN (2016). The effect of latency reversal agents on primary CD8^+^ T cells: Implications for shock and kill strategies for human immunodeficiency virus eradication. EBioMedicine.

[27] Covino DA, Desimio MG, Doria M (2021). Combinations of histone deacetylase inhibitors with distinct latency reversing agents variably affect HIV reactivation and susceptibility to NK cell-mediated killing of T cells that exit viral latency. Int. J. Mol. Sci..

[28] Giuliani E, Desimio MG, Doria M (2019). Hexamethylene bisacetamide impairs NK cell-mediated clearance of acute T lymphoblastic leukemia cells and HIV-1-infected T cells that exit viral latency. Sci. Rep..

[29] Ni L (2017). The histone deacetylase inhibitor valproic acid inhibits NKG2D expression in natural killer cells through suppression of STAT3 and HDAC3. Sci. Rep..

[30] Lanier LL (2015). NKG2D receptor and its ligands in host defense. Cancer. Immunol. Res..

[31] Desimio MG, Giuliani E, Doria M (2017). The histone deacetylase inhibitor SAHA simultaneously reactivates HIV-1 from latency and up-regulates NKG2D ligands sensitizing for natural killer cell cytotoxicity. Virology.

[32] Desimio MG, Covino DA, Doria M (2019). Potential of the NKG2D/NKG2DL axis in NK cell-mediated clearance of the HIV-1 reservoir. Int. J. Mol. Sci..

[33] Pili R (2012). Phase I study of the histone deacetylase inhibitor entinostat in combination with 13-cis retinoic acid in patients with solid tumours. Br. J. Cancer.

[34] Wightman F (2013). Entinostat is a histone deacetylase inhibitor selective for class 1 histone deacetylases and activates HIV production from latently infected primary T cells. AIDS.

[35] Wei DG (2014). Histone deacetylase inhibitor romidepsin induces HIV expression in CD4 T cells from patients on suppressive antiretroviral therapy at concentrations achieved by clinical dosing. PLoS Pathog..

[36] Martínez-Bonet M (2015). Synergistic activation of latent HIV-1 expression by novel histone deacetylase inhibitors and bryostatin-1. Sci. Rep..

[37] Ogbomo H, Michaelis M, Kreuter J, Doerr HW, Cinatl J (2007). Histone deacetylase inhibitors suppress natural killer cell cytolytic activity. FEBS Lett..

[38] Beck J (2005). Phase I pharmacokinetic (PK) and pharmacodynamic (PD) study of oral LBH589B: a novel histone deacetylase (HDAC) inhibitor. J. Clin. Oncol..

[39] Archin NM (2009). Expression of latent HIV induced by the potent HDAC inhibitor suberoylanilide hydroxamic acid. AIDS Res. Hum. Retroviruses.

[40] Desimio MG, Giuliani E, Ferraro AS, Adorno G, Doria M (2018). In vitro exposure to prostratin but not bryostatin-1 improves natural killer cell functions including killing of CD4(+) T cells harboring reactivated human immunodeficiency virus. Front. Immunol..

[41] Gavegnano C (2017). Novel mechanisms to inhibit HIV reservoir seeding using Jak inhibitors. PLoS Pathog..

[42] Tremblay-McLean A, Coenraads S, Kiani Z, Dupuy FP, Bernard NF (2019). Expression of ligands for activating natural killer cell receptors on cell lines commonly used to assess natural killer cell function. BMC Immunol..

[43] Jones RB (2016). A subset of latency-reversing agents expose HIV-infected resting CD4^+^ T-cells to recognition by cytotoxic T-lymphocytes. PLoS Pathog..

[44] Curreli F, Ahmed S, Victor SMB, Debnath AK (2020). Identification of combinations of protein kinase C activators and histone deacetylase inhibitors that potently reactivate latent HIV. Viruses.

[45] Zhu S (2015). The narrow-spectrum HDAC inhibitor entinostat enhances NKG2D expression without NK cell toxicity, leading to enhanced recognition of cancer cells. Pharm. Res..

[46] Mota TM (2020). Integrated assessment of viral transcription, antigen presentation, and CD8(+) T cell function reveals multiple limitations of Class I-selective histone deacetylase inhibitors during HIV-1 latency reversal. J. Virol..

[47] Pardons M, Fromentin R, Pagliuzza A, Routy JP, Chomont N (2019). Latency-reversing agents induce differential responses in distinct memory CD4 T cell subsets in individuals on antiretroviral therapy. Cell. Rep..

[48] Bullen CK, Laird GM, Durand CM, Siliciano JD, Siliciano RF (2014). New ex vivo approaches distinguish effective and ineffective single agents for reversing HIV-1 latency in vivo. Nat. Med..

[49] Larragoite ET (2022). Histone deacetylase inhibition reduces deleterious cytokine release induced by ingenol stimulation. Biochem. Pharmacol..

[50] Banga R, Procopio FA, Cavassini M, Perreau M (2015). In vitro reactivation of replication-competent and infectious HIV-1 by histone deacetylase inhibitors. J. Virol..

[51] Grau-Expósito J (2019). Latency reversal agents affect differently the latent reservoir present in distinct CD4^+^ T subpopulations. PLoS Pathog..

[52] Shan L (2012). Stimulation of HIV-1-specific cytolytic T lymphocytes facilitates elimination of latent viral reservoir after virus reactivation. Immunity.

[53] Burnett JC (2010). Combinatorial latency reactivation for HIV-1 subtypes and variants. J. Virol..

[54] Laird GM (2015). Ex vivo analysis identifies effective HIV-1 latency-reversing drug combinations. J. Clin. Invest..

[55] Reuse S (2009). Synergistic activation of HIV-1 expression by deacetylase inhibitors and prostratin: Implications for treatment of latent infection. PLoS ONE.

[56] Mishra A, Sullivan L, Caligiuri MA (2014). Molecular pathways: Interleukin-15 signaling in health and in cancer. Clin. Cancer Res..

[57] Williams SA (2004). Prostratin antagonizes HIV latency by activating NF-kappaB. J. Biol. Chem..

[58] Medler TR (2016). HDAC6 deacetylates HMGN2 to regulate Stat5a activity and breast cancer growth. Mol. Cancer. Res..

[59] Anderson I (2014). Heat shock protein 90 controls HIV-1 reactivation from latency. Proc. Natl. Acad. Sci. USA.

[60] Bali P (2005). Inhibition of histone deacetylase 6 acetylates and disrupts the chaperone function of heat shock protein 90: A novel basis for antileukemia activity of histone deacetylase inhibitors. J. Biol. Chem..

[61] Cerboni C (2007). Human immunodeficiency virus 1 Nef protein downmodulates the ligands of the activating receptor NKG2D and inhibits natural killer cell-mediated cytotoxicity. J. Gen. Virol..

[62] Fogli M (2008). Lysis of endogenously infected CD4^+^ T cell blasts by rIL-2 activated autologous natural killer cells from HIV-infected viremic individuals. PLoS Pathog..

[63] Marras F (2017). Control of the HIV-1 DNA reservoir is associated in vivo and in vitro with NKp46/NKp30 (CD335 CD337) inducibility and interferon gamma production by transcriptionally unique NK cells. J. Virol..

[64] Matusali G, Potestà M, Santoni A, Cerboni C, Doria M (2012). The human immunodeficiency virus type 1 Nef and Vpu proteins downregulate the natural killer cell-activating ligand PVR. J. Virol..

[65] Tomescu C, Mavilio D, Montaner LJ (2015). Lysis of HIV-1-infected autologous CD4^+^ primary T cells by interferon-alpha-activated NK cells requires NKp46 and NKG2D. AIDS.

[66] Garrido, C. *et al*. Interleukin-15-stimulated natural killer cells clear HIV-1-infected cells following latency reversal ex vivo. *J. Virol.* **92**, e00235-18. doi: 10.1128/JVI.00235-18 (2018) 10.1128/JVI.00235-18 PMC5974478 29593039

[67] Marsden MD (2017). In vivo activation of latent HIV with a synthetic bryostatin analog effects both latent cell "kick" and "kill" in strategy for virus eradication. PLoS Pathog..

[68] Yang H (2019). Dual effects of the novel ingenol derivatives on the acute and latent HIV-1 infections. Antiviral Res..

