# Supplementary material for: Impact of IL-15 and latency reversing agent combinations in the reactivation and NK cell-mediated suppression of the HIV reservoir
Source: Sci Rep. 2022 Nov 3;12:18567. doi: 10.1038/s41598-022-23010-5 (PMC9633760; doi:10.1038/s41598-022-23010-5)
Supplement: Supplementary file 1 — Supplementary Figure S1. [file 41598_2022_23010_MOESM1_ESM.pdf]

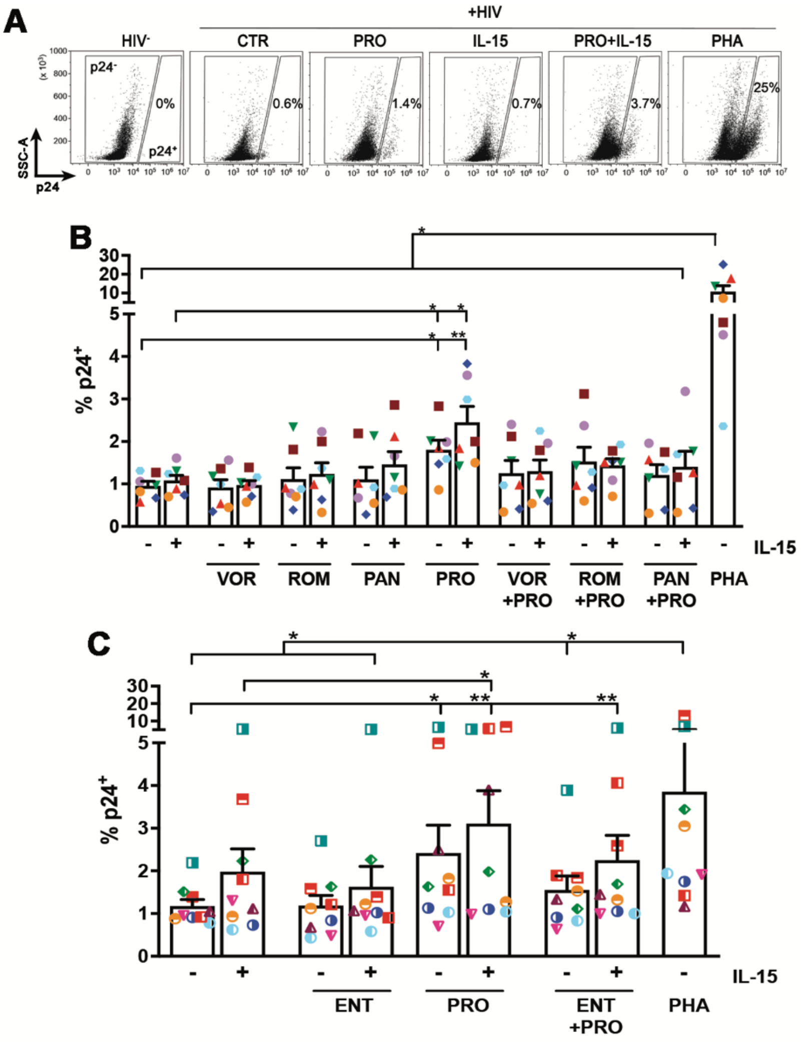

Supplementary Fig. S1. Effect of IL-15 addition to HDACis or PRO alone and in combination on reactivation of HIV-1 latently infected CD4$^+$ T cells. Latently HIV-infected CD4$^+$ T cell cultures were established as described in Materials and Methods then, at day 3 post-infection, cells were not stimulated (CTR) or stimulated with IL-15, PHA, HDACi, or PRO alone or in HDACi+PRO combinations with or without addition of IL-15. After 48 h, cells were washed and the same stimuli were added again. After 18 h, the expression of intracellular p24 was analyzed by flow cytometry. **(A)** Representative dot plots show the frequency of p24$^+$ cells in control (CTR), PRO-, IL-15- and PRO+IL15- and PHA-stimulated cultures gated by setting non-infected cells (HIV$^-$) at 0%. **(B-C)**. Bars show mean ± SEM values obtained in experiments performed with 16 different donors each one represented with a distinctive symbol (7 donors for pan-HDACi in panel B, 9 donors for ENT combinations in panel C). Statistic was performed using paired Wilcoxon test. *$p < 0.05$; **$p < 0.01$.
