# Supplementary material for: Impact of IL-15 and latency reversing agent combinations in the reactivation and NK cell-mediated suppression of the HIV reservoir
Source: Sci Rep. 2022 Nov 3;12:18567. doi: 10.1038/s41598-022-23010-5 (PMC9633760; doi:10.1038/s41598-022-23010-5)
Supplement: Supplementary file 2 — Supplementary Figure S2. [file 41598_2022_23010_MOESM2_ESM.pdf]

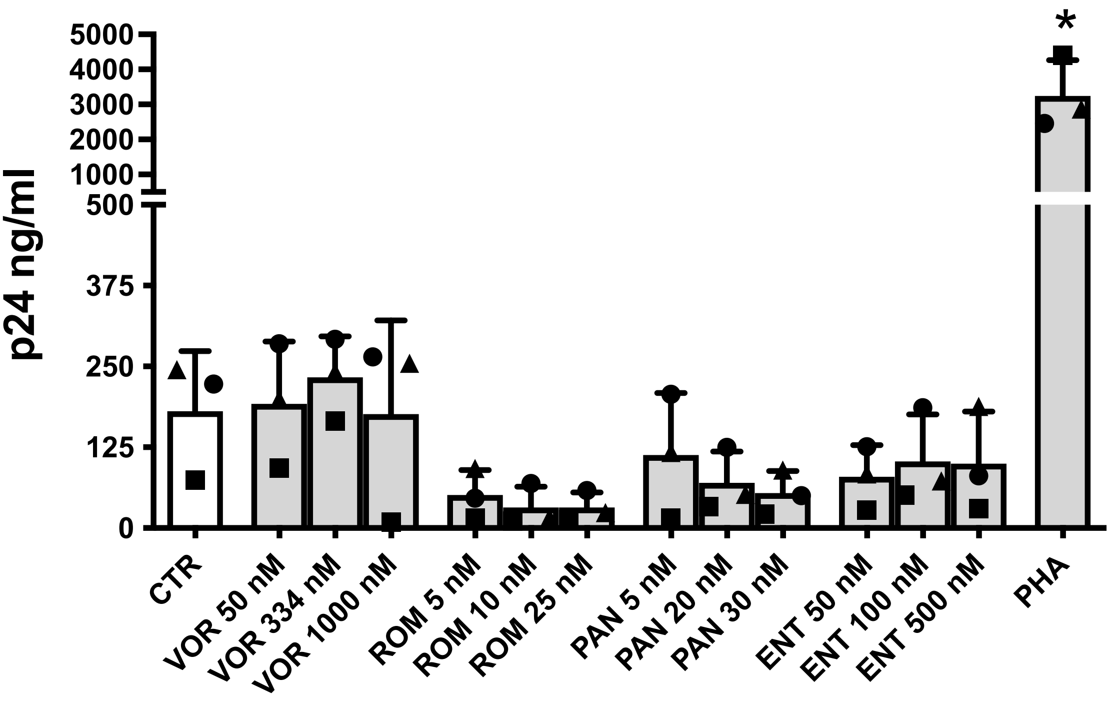

Supplementary Fig. S2. Effect of increasing HDACi concentrations on HIV reactivation in latently infected CD4$^+$ T cells. Latent HIV infection was established in resting CD4$^+$ T cells derived from healthy donors as described in Materials and Methods. Three days post-infection cells were collected and replaced in culture in medium alone (CTR) or supplemented with three doses of HDACi (50, 334, 1000 nM VOR; 5, 10, 25 nM ROM; 5, 20, 30 nM PAN; 50, 100, 500 nM ENT) or with 10 µg/ml PHA. After 48 h cells were washed and seeded at 2×10^6/ml in the same initial conditions. Five days later, p24 released in the culture medium was analyzed by ELISA. Bars represent mean ± SEM obtained from 3 independent donors. Comparisons were performed using paired Wilcoxon versus unstimulated control; *$p < 0.05$.
